# Radiomics and deep learning methods for the prediction of 2-year overall survival in LUNG1 dataset

**DOI:** 10.1038/s41598-022-18085-z

**Published:** 2022-08-19

**Authors:** Anna Braghetto, Francesca Marturano, Marta Paiusco, Marco Baiesi, Andrea Bettinelli

**Affiliations:** 1 Physics and Astronomy Department “Galileo Galilei”, University of Padova, Via Marzolo 8, 35131 Padua, Italy; 2 INFN, Sezione di Padova, Via Marzolo 8, 35131 Padua, Italy; 3 Medical Physics Department, Veneto Institute of Oncology-IOV IRCCS, Padua, Italy; 4 Department of Information Engineering, University of Padova, Padua, Italy

**Keywords:** Biomedical engineering, Cancer, Predictive markers, Computer science, Image processing, Machine learning

## Abstract

In this study, we tested and compared radiomics and deep learning-based approaches on the public LUNG1 dataset, for the prediction of 2-year overall survival (OS) in non-small cell lung cancer patients. Radiomic features were extracted from the gross tumor volume using Pyradiomics, while deep features were extracted from bi-dimensional tumor slices by convolutional autoencoder. Both radiomic and deep features were fed to 24 different pipelines formed by the combination of four feature selection/reduction methods and six classifiers. Direct classification through convolutional neural networks (CNNs) was also performed. Each approach was investigated with and without the inclusion of clinical parameters. The maximum area under the receiver operating characteristic on the test set improved from 0.59, obtained for the baseline clinical model, to 0.67 ± 0.03, 0.63 ± 0.03 and 0.67 ± 0.02 for models based on radiomic features, deep features, and their combination, and to 0.64 ± 0.04 for direct CNN classification. Despite the high number of pipelines and approaches tested, results were comparable and in line with previous works, hence confirming that it is challenging to extract further imaging-based information from the LUNG1 dataset for the prediction of 2-year OS.

## Introduction

Lung cancer is one of the most aggressive cancer types with a 5-year relative survival rate of only 19%^1^. The main causes of the disease are attributable to bad habits (e.g. smoking and drinking), adverse circumstances like exposure to noxious or radioactive materials (e.g. radon, asbestos), recurring lung inflammation, or lung scarring secondary to tuberculosis^2^.

Lung cancer can be classified into two main categories: non-small cell lung cancer (NSCLC)^2^ and small cell lung cancer^3^. NSCLC alone accounts for more than 85% of lung cancer cases and can be further subdivided into two types: non-squamous carcinoma (including adenocarcinoma, large-cell carcinoma, and other cell types) and squamous cell epidermoid carcinoma.

Accurate diagnosis and staging are fundamental components to optimize the therapy and achieve a good prognosis^4^. In this context, biomedical imaging, such as magnetic resonance (MR), computed tomography (CT), or positron emission tomography (PET), plays a pivotal role, offering several non-invasive modalities for the high-resolution three-dimensional visualization and characterization of the lesion. The availability of high-quality digital images has determined significant improvements in cancer diagnosis and has opened new frontiers for the application of radiomics and artificial intelligence (AI) techniques.

In oncology, radiomics provides a noninvasive way to capture and quantitatively describe tumour characteristics from radiological medical imaging^5^. Radiomics consists in the high-throughput extraction and analysis of quantitative features from a region of interest (ROI) with the aim of building predictive models of clinical endpoints (e.g. histological outcomes, survival time, tumor stage, malignancy vs. benignity) that may assist physicians in clinical decision making. Handcrafted radiomic features have demonstrated encouraging results^6^ in cancer research and AI-based techniques, in particular deep learning (DL) approaches, have also proved to be valuable tools for the automatic learning of potentially relevant patterns from medical images^7,8^, which could further improve the accuracy of radiomic models.

A pioneering work in radiomics was performed by Aerts et al.^9^ who built a prognostic signature valid for both lung and head & neck cancers using the public LUNG1 dataset collected at the MAASTRO Clinic, The Netherlands. They extracted a total of 440 radiomic features quantifying tumor intensity, shape, and texture from CT images and developed a Cox proportional-hazard model to predict the survival time, obtaining a validation concordance index (C-index) of 0.65 and 0.69 for lung and head and neck cancers, respectively.

After the study, in the context of FAIR^10^ (findable, accessible interoperable and reusable) data, LUNG1 dataset has been published on the data repository platform “The Cancer imaging archive”—TCIA^11^, hence paving the way for several studies that further explored the predictive power of this dataset.

To the best of our knowledge, ten studies used the public LUNG1 dataset to develop AI-based models: nine studies applied standard radiomic analysis for clinical outcome prediction (2 of which infer the 2-year OS^12,18^, and 1 applies DL methods for survival outcome prediction^17^), while one introduced DL for tumor segmentation and applied machine learning methods to test the Aerts’ radiomic signature for different GTV delineations^20^. These works have been summarized in Table 1.

**Table 1:** Summary of previous studies based on LUNG1 public dataset.

Work	Aim	Approach	Lung dataset	Methods	Results	Conclusions	RQS (%)
Parmar et al., 2015^12^	Prediction of 2-year OS	Radiomic features	• LUNG1 for training • LUNG2 for validation	Different feature selection methods and ML classifiers	Highest average AUC for Wilcoxon test based feature selection method (AUC = 0.65) and a random forest classifier (AUC = 0.66)	The choice of classification method is the most dominant source of performance variation	31
Parmar et al., 2015^13^	Investigation of the clinical relevance of radiomic clusters	Radiomic features	• LUNG1 for training • LUNG2 for validation	• Cluster analysis • Cox Proportional Hazards model on cluster centroids	• All lung clusters were significantly associated to survival • AUC = 0.64 for tumour histology and tumour stage prediction	Clustering and prognostic characteristics of radiomic features are cancer-specific	22
Wu et al., 2016^14^	Classification of tumour histologic subtypes	Radiomic features	• LUNG1 for training • LUNG2 for validation	Different feature selection methods and ML classifiers	Naive Bayes classifier combined with ReliefF achieved the highest AUC of 0.72	Radiomic features show significant association with the lung tumour histology	28
Lambrecht et al., 2017^15^	Classification of: T-stage, overall-stage, N-stage, M-stage, histology; Prediction of survival time	Radiomic features	LUNG1 for training and validation	K-means clustering Random Forest and Neural Networks	Neural networks achieved the highest ACC of 63.9%	Results are highly dependent on the choice of the clinical outcome to predict	33
Chaddad et al., 2017^16^	Prediction of the survival outcome for different cancer subtype and stage groups	Radiomic features	LUNG1 for training and validation	Random Forest classifier	Highest AUC of 0.76 for the TNM *stage I* group	Radiomic features can be used as indicators of survival for large-cell carcinoma patients with primary tumour size and no lymph-node metastasis	39
Haarburger et al., 2018^17^	Prediction of survival outcome	• Radiomic & deep features + ML • Direct CNN prediction	LUNG1 for training and validation	• Cox Proportional Hazards model • CNN Hazard model	• C-index of 0.623 for model fitted with selected radiomic and CNN features • C-index of 0.585 for CNN direct hazard prediction	Cox models with radiomics and deep features outperform CNNs with concatenated radiomics features	31
Shi et al., 2019^18^	Prediction of 2-year OS and survival outcome with Aerts radiomic signature	Radiomic features	• LUNG1 for training • LUNG2 for validation	Multivariable logistic regression and Cox Proportional Hazards model	AUC of 0.61 and Harrell C-index of 0.58 on LUNG2 dataset	External validation of radiomic models can be done with decentralized data without exchanging patients’ sensitive data	28
Welch et al., 2019^19^	Prediction of survival outcome depending on several factors using Aerts radiomic signature	Radiomic features	• LUNG1 for training • H&N1 for validation	Cox Proportional Hazards model	• C-index of 0.64 on H&N1 external dataset • Tumour volume was as prognostic as the radiomic signature in H&N1 (C-index = 0.64)	The radiomic signature was a surrogate for tumour volume	28
Haarburger et al., 2020^20^	Testing of the Aerts radiomic signature for different GTV delineations	Radiomic features	LUNG1 for training and validation	• ICC for feature stability • Cox Proportional Hazards model	• 28.7% of all features had an ICC < 0.9 • C-indices of Cox models varied between 0.57 and 0.58	Features are subject to higher (GLRLM and GLSZM) and lower (shape, GLCM, and NGTDM) variance across delineations	25
Ubaldi et al., 2021^21^	Classification of tumor histology and overall stage (I or II)	Radiomic features	LUNG1 for training and validation on an private dataset and viceversa + Merging of the two datasets	Different feature selection methods and ML classifiers	• AUC = 0.72 for histology classification with merged datasets • AUC = 0.84 when training on LUNG1 and testing on another dataset	Histology classification improved when considering subjects with overall stages I and II, hence reducing the heterogeneity of the sample	31

*ACC* accuracy, *AUC* area under the receiver operating characteristic curve, *C-index* concordance index, *CNN* convolutional neural network, *GLCM* grey level co-occurrence matrix, *GLRLM* grey level run length matrix, *GLSZM* grey level size zone matrix, *ICC* intra-class correlation coefficient, *ML* machine learning, *NGTDM* neighboring grey tone difference matrix, *OS* overall survival,* RQS* radiomic quality score.

In this work, we investigate and compare a wide set of methodologies for the prediction of the 2-year OS in NSCLC considering patients from the public LUNG1 dataset^11^. Firstly, we evaluated the baseline model built with clinical data, then we implemented two main approaches to predict the 2-year OS, one feature-based and one CNN-based. In the feature-based approach, several machine-learning models and feature selection methods were fed either with hand-crafted radiomic features, with deep features (extracted through a convolutional autoencoder—CAE), or with the combination of the two feature sets. The CNN-based approach instead directly predicted the survival outcome from the image, without the need of a separate feature extraction step. All methods and approaches were also tested with the inclusion of clinical data.

The inclusion of DL methods into our analysis assessed whether they could introduce margins of improvement in the prediction of the 2-year OS.

## Materials and methods

### Dataset

We resorted to the LUNG1 dataset, consisting of 422 patients with inoperable NSCLC treated at MAASTRO Clinic, The Netherlands, with radical radiotherapy or chemo-radiation. The LUNG1 dataset is publicly available for download, thus institutional review board approval was not required for this study. All methods concerning the acquisition and usage of this dataset were in accordance with relevant guidelines and regulations.

All patients underwent an FDG PET-CT scan for radiotherapy treatment planning. A spiral CT, with a 3 mm slice thickness, was performed covering the thoracic region. Gross tumor volume (GTV) segmentations were delineated by a radiation oncologist on fused FDG PET-CT images. Details on the protocol can be found in a previous work^9^.

**Figure 1:**
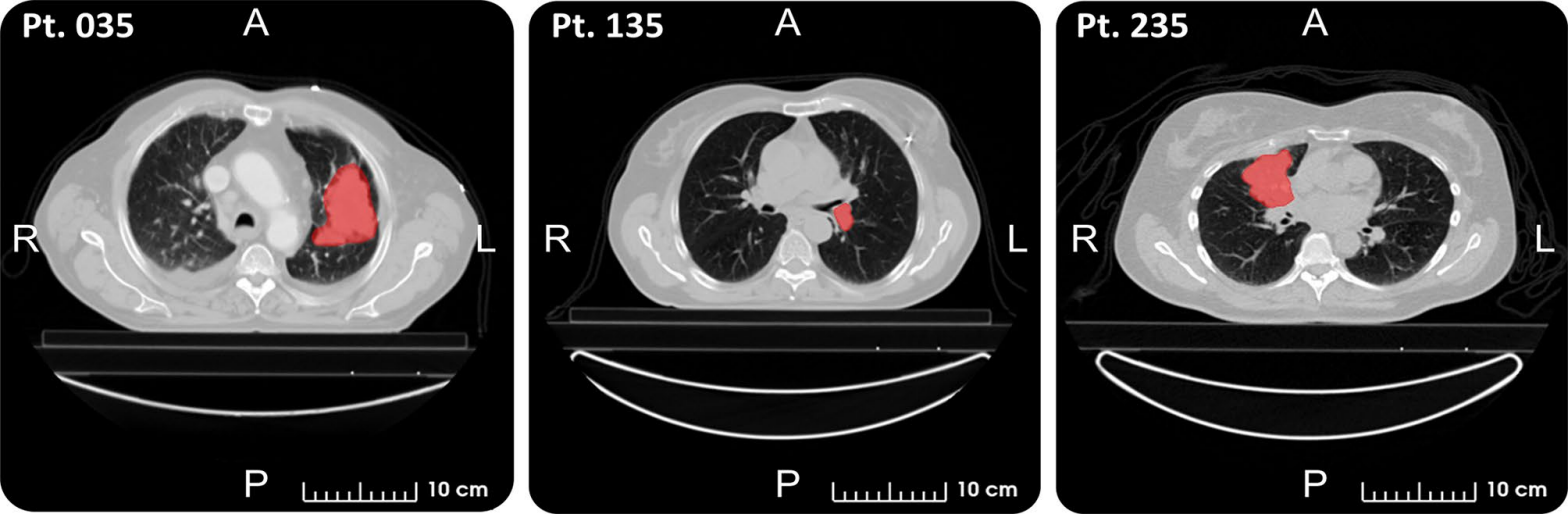
Representative slices of three LUNG1 patients with superimposed delineation of the GTV (viewing window: [−1000, 400] HU).

The public dataset comprehends, for each patient, seven clinical parameters (i.e., sex, age at diagnosis, TNM staging, AJCC staging, histology, survival time, and death status), a chest CT image, and a manual delineation of the GTV. After the exclusion of 11 patients, we considered a total of 411 patients for this study. Exclusion criteria were: patients lost to follow-up prior to 2 years (one case), error in the GTV file (two cases), missing segmentation (three cases), and misalignment of tumor segmentation (five cases). Figure 1 shows a representative CT slice of three LUNG1 patients with the superimposed delineation of the GTV.

Regarding the clinical information, we used survival time and death status event to derive survival labels, whereas we considered sex, age at diagnosis, TNM staging, AJCC staging and histology as the clinical features for the OS prediction.

### Pipelines of analysis

For the analysis conducted in this study, we resorted to two different approaches. The first was a feature-based approach and considered clinical, radiomic, deep features and all combinations of the above.

Features were then post-processed with four feature selection/dimensionality reduction algorithms and fed to six binary classifiers, for a total of 24 different pipelines, to predict the probability that the patients survived more than two years from the diagnosis.

The second approach consisted in the direct classification of the patients with CNNs and considering both 2D and 2.5D architectures.

**Figure 2:**
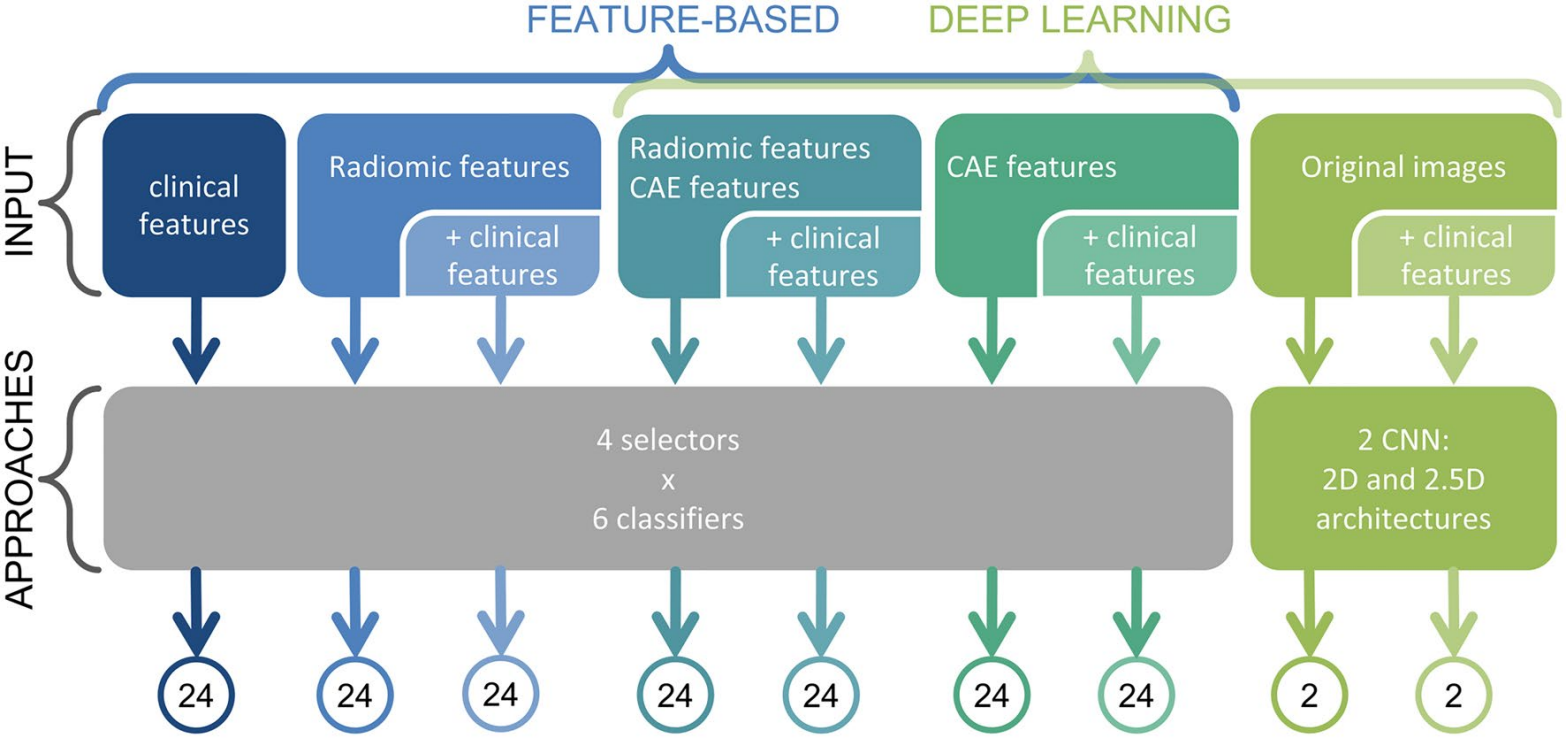
Flow chart of the analysis for the two different classification approaches. In the figure, the number of tested models for each approach is also visible. A total of 168 different pipelines were tested for the feature-based approach (including clinical, radiomic and deep features only and their combination), while 4 architectures were tested for the CNN-based approach.

Each approach considered features and/or images both alone and in combination with clinical parameters. In Fig. 2, the flow chart of the analysis is shown for the aforementioned approaches.

The performance of the 2-year OS prediction for each approach was measured through the area under the receiver operating characteristic curve (AUC) that relates the true positive rate to the false positive rate achieved by each classifier. The entire dataset was randomly divided five times into training and test sets (ratio 3:1), with each subdivision called shuffle split. For each shuffle split, 25% of the data was used as a test set, while the remaining 75% was used for model training. On the training set, we performed an inner cross-validation procedure to tune the hyper-parameters of the corresponding model (e.g., the architecture of the neural network). Then, the performance of the model with optimized hyper-parameters was evaluated on the test set. The procedure was repeated for the five different outer splits of the initial dataset and the average AUCs obtained on the test set of each split were finally calculated. For the pipelines with the highest average test AUC we also reported the standard deviation over the five shuffle splits.

### Feature-based approach

In the feature-based approach, the 2-year OS prediction was carried out using three different sets of features (clinical, radiomic and deep features) and all their combinations. Eventually 168 pipelines were tested for the feature-based approach (Fig. 2).

#### Radiomic feature extraction

Radiomic features extraction was performed with the public Pyradiomics library (version 3.0.1)^22^ after isotropic interpolation to 1 × 1 × 1 mm, and resegmentation of the ROI binary mask in the range [−600, 400]. The radiomic feature set included shape, first order, and textural features (i.e., grey level co-occurrence matrix—GLCM, grey level run length matrix—GLRLM, grey level size zone matrix—GLSZM, grey level distance zone matrix—GLDZM, and the neighboring grey tone difference matrix—NGTDM), which were computed on the entire tumor volume. To extract additional information from the ROI, four different filtering techniques were applied to the original images (i.e., wavelet, square root, gradient magnitude, and a Laplacian of Gaussian). In particular, for the original images, a fixed bin size discretization approach was used (bin width of 25 HU), whereas for filtered images a fixed bin number approach was employed (32 bins), as suggested by the Image Biomarker Standardization Initiative (IBSI)^23^. Textural features were aggregated in three dimensions (3D:mrg was the method of choice for rotation-dependent textural features).

Eventually, for each lesion, considering both original and filtered images, we obtained a total of 1118 radiomic feature values for the characterization of the tumor phenotype. As a preliminary step, only features that were linearly independent from each other (i.e., Pearson correlation coefficient ≤ 0.95) were kept for subsequent analysis. Additional details about radiomic feature extraction can be found in the supplementary material (Sect. 1.1).

#### Deep feature extraction

For deep feature calculation, we used a common architecture, the convolutional autoencoder, which compresses the information enclosed within the image into a set of latent variables^7,24,25^. The common structure of a CAE is made of a stacked encoder and decoder: the encoder translates the input image into a set of latent variables that summarise the relevant information contained within the image; subsequently, the decoder reconstructs the original image from the latent variables.

For the analysis, we resorted to bi-dimensional architecture to reduce model complexity and the risk of overfitting. For each patient, we considered five bi-dimensional slices: the one with the largest cross-sectional area of the ROI and the four adjacent slices. Afterward, the dataset was augmented by applying eight affine transformations to the original images, i.e., four rotations (i.e., 5°, 10°, 15°, 20°) and four translations (± 5 voxel in the anteroposterior and lateral direction). This process increased the training set size from 1540 samples (5 slices for each patient in the training set) to 13,860 images (45 slices per patient). Eventually, both original and augmented images were cropped around the ROI to obtain an image dimension of 64 × 64 voxels, which allowed both to reduce the computational cost for network training and to be consistent with the radiomic analysis which focuses on the segmented lesion only. This procedure guarantees a robust comparison between the two methods. For each slice, a total of 500 deep features were obtained with the considered method.

The whole analysis was implemented through the Python *Keras* library, considering a fixed architecture for the CAE. Details about the structure can be found in the supplementary material (Sect. 3.1).

#### Feature reduction/classification

To reduce the chance of overfitting, the number of features used for the classification task was reduced to 5, 10, 20, or 40 by employing two classes of methods: (a) feature selection and (b) dimensionality reduction techniques. The first class selects the optimal set of features by looking at their quantitative description (e.g., their variance or importance within a model) to improve classification accuracy. Feature selection methods used in this work were ANOVA F-value and SelectFromModel (SFM) methods. The second class of methods eliminates redundancy among variables, for example by mapping the original features to a space of lower dimension. Dimensionality reduction methods were principal component analysis (PCA) and feature clustering. Additional details on feature selection/reduction methods can be found in supplementary material (Sect. 2.1).

Once features were selected (or reduced), the prediction of the 2-year OS was carried out with six different classifiers, i.e., support vector machines (SVM), bagging (BAG), random forests (RF), extreme gradient boosting (XGB), feed-forward neural networks (NNET) and k-nearest neighbors (NN). Additional details about the classifiers are reported in supplementary material (Sect. 2.2).

By pairing each feature selection/reduction method with each classifier, we obtained 24 different pipelines. The hyper-parameters of each pipeline (i.e., number of features to be included in the model, kernel and regularization term for SVM, number of trees for BAG, RF and XGB, NNET architecture, number of neighbors for NN) were tuned using a grid search approach implemented with a stratified 2-repeated threefold cross-validation (CV) procedure on the training data of each shuffle split. Once the model with the optimal hyper-parameters was selected in the training set with CV, it was applied to the test set. Eventually, results obtained for each shuffle split were averaged to derive the reported model performances.

### CNN-based approach

The last approach consisted in the application of CNNs to directly classify the bi-dimensional CT slices of the tumor mass. A CNN presents a feed-forward architecture able to learn relevant information enclosed within images to predict an outcome of interest. CNNs are made of an input layer (e.g., for the input image), an output layer and several hidden layers in between made of convolutional, pooling and fully-connected layers.

Two different CNN models were tested in this work, i.e., 2D-CNN applied to the original bi-dimensional slices of each image and 2.5D-CNN applied to 5-channel images (where each channel corresponded to one of the five bi-dimensional CT slices adjacent to the slice with the largest cross-sectional tumor area). Image pre-processing and data augmentation were performed in the same way as the deep features approach. For both models, training with the inclusion of clinical features was also considered. The whole analysis was implemented through Python *Keras* library and, for each CNN, hyper-parameters corresponding to the regularization terms (i.e., dropout rate and L1 and L2 weight decays), were tuned through a grid search approach in a stratified 2-repeated threefold cross-validation implemented in each shuffle split. Details about CNN architectures and grid search parameters can be found in supplementary material (Sect. 3.2).

## Results

### Results for feature-based approach

#### Clinical features

**Figure 3:**
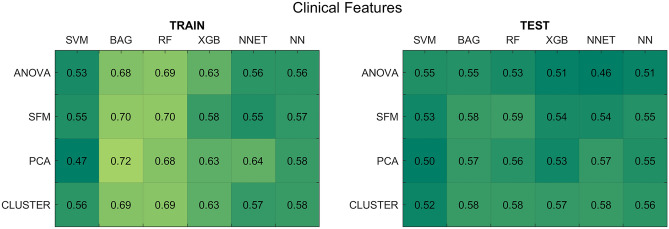
Results for the clinical feature-based models. (Left panel) Average AUCs on the five training splits. (Right panel) Average AUCs on the test splits. *SFM* SelectFromModel, *PCA* principal component analysis, *CLUSTER* feature agglomeration through clustering, *SVM* support vector machines, *BAG* bagging, *RF* random forest, *XGB* extreme gradient boosting, *NNET* neural network, *NN* k-nearest neighbours.

For the models trained with clinical data, the average AUCs across the five shuffle splits are reported in Fig. 3. The average prediction results on the test set did not exceed 0.59, thus suggesting that clinical features do not carry enough information for the accurate prediction of the 2-year OS.

#### Radiomic features

**Figure 4:**
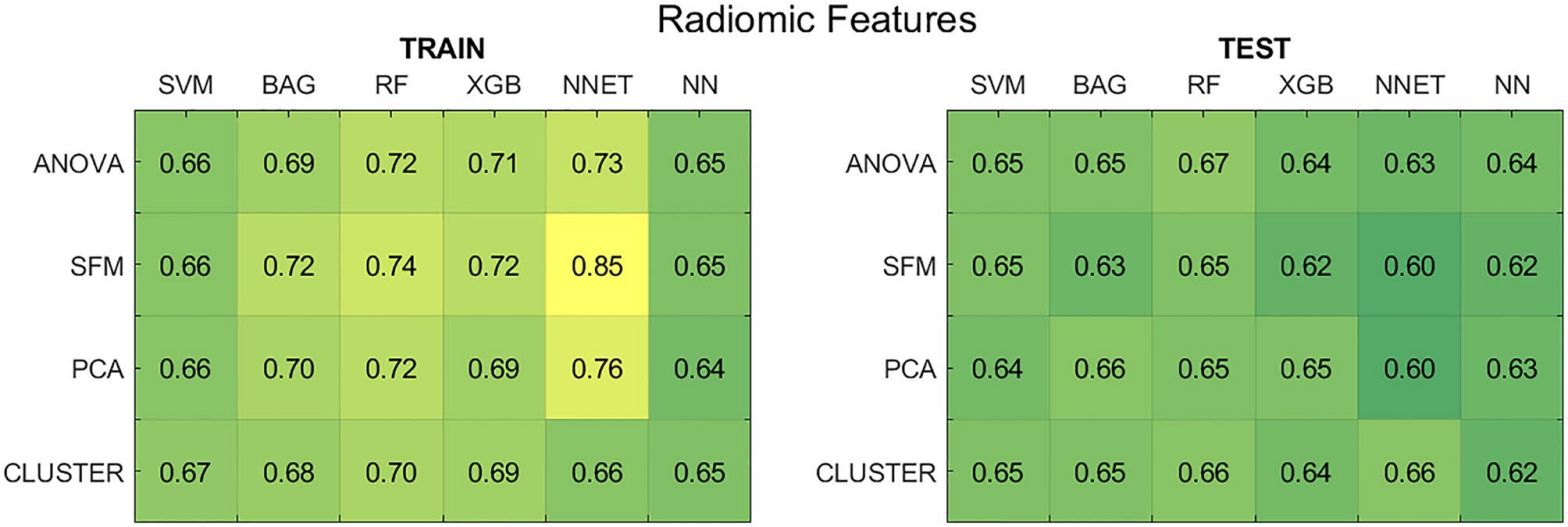
Results for the radiomic feature-based models without the use of clinical data. (Left panel) Average AUCs on the five training splits. (Right panel) Average AUCs on the test splits. *SFM* SelectFromModel, *PCA* principal component analysis, *CLUSTER* feature agglomeration through clustering, *SVM* support vector machines, *BAG* bagging, *RF* random forest, *XGB* extreme gradient boosting, *NNET* neural network, *NN* k-nearest neighbours.

Figure 4 shows the average AUCs for the radiomic feature-based approach. The results refer to the pipelines trained with the best hyper-parameters, without the inclusion of clinical data (results with clinical data are reported in Supplementary Fig. S4).

By comparing the results of the training and test set, significant differences in performance are visible for some classification pipelines. The SFM method in combination with the NNET shows the highest training AUC (> 0.80), but also the worst test AUC (∼0.60), both with and without including clinical data (supplementary Fig. S4). For this pipeline, SFM identified a number of forty radiomic features to be fed to the classifier on three shuffle splits out of five, thereby inserting too many variables in the model and reducing the ability of the NNET to generalize on unseen data. The same issue was observed when SFM was combined with ensemble classification methods (i.e., BAG, RF, and XGB). Afresh, a high number of features was selected for these pipelines, exceeding 20 for all splits. At the same time, SFM in combination with SVM and NN classifiers did not show any considerable gap between training and test set results attributable to overfitting (train/test AUC of 0.66/0.65 for SVM and 0.65/0.62 for NN). This suggests that the performance of the SFM method was highly dependent on the classifier, thus not ensuring proper generalization across all pipelines.

In contrast, when the NNET was coupled with CLUSTER, training and test AUC became comparable (average AUC around 0.66 across all splits). In this case, in 4 out of 5 splits, only five radiomic features were selected, hence allowing more parsimonious and generalizable models. Nevertheless, as visible from Fig. 4, the NNET classifier presented the worst generalization ability, achieving the highest train-test performance gap for some pipelines. However, the highest performance over the test set was achieved by RF classifier in combination with the ANOVA feature selection method. In both the analyses with and without clinical data, the training AUC reached 0.72 ± 0.05, while the test AUC reached 0.67 ± 0.03. Therefore, this pipeline obtained the best compromise between performance and generalization ability.

#### Deep features

**Figure 5:**
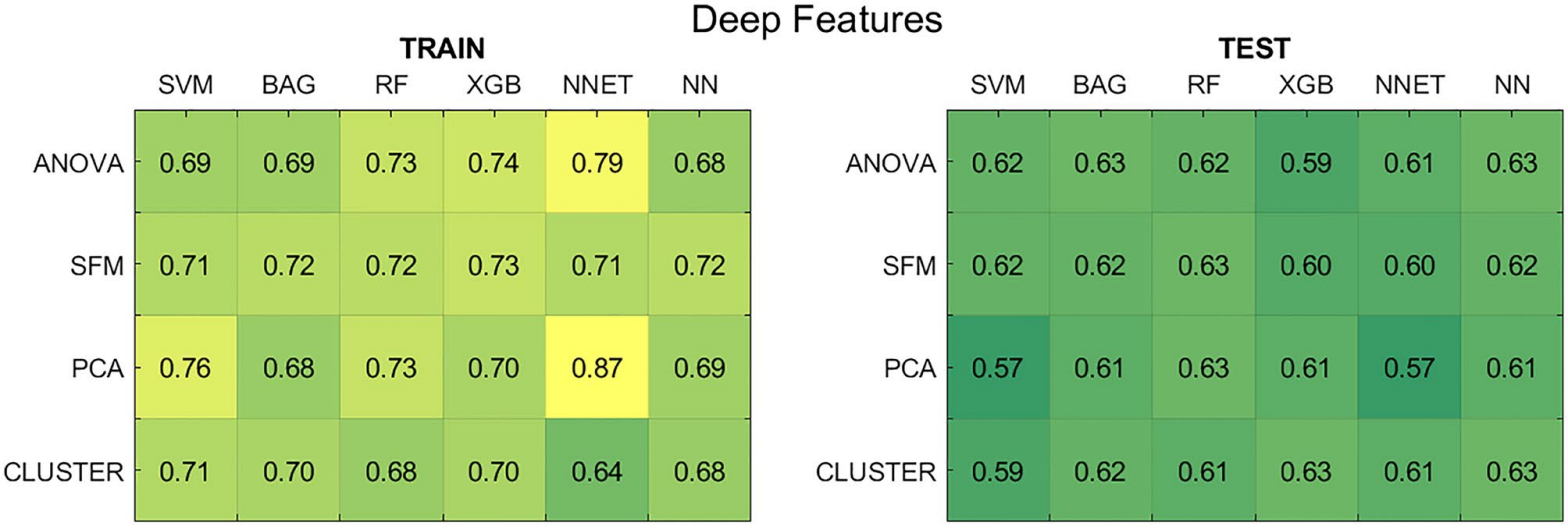
Results for the deep feature-based models without clinical data. (Left panel) Average AUCs on the five training splits. (Right panel) Average AUCs on the test splits. *SFM* SelectFromModel, *PCA* principal component analysis, *CLUSTER* feature agglomeration through clustering, *SVM* support vector machines, *BAG* bagging, *RF* random forest, *XGB* extreme gradient boosting, *NNET* neural network, *NN* k-nearest neighbours.

Figure 5 reports the average AUCs across the five shuffle splits for deep feature-based models, considering the optimal hyper-parameters for each pipeline, for the case in which clinical data were not considered in the feature set (results with the addition of clinical data are reported in Supplementary Fig. S5).

For the deep feature approach, the SVM classifier showed the poorest performances both on the training and test sets. The pipeline formed by NNET classifier and PCA presented the highest divergence between training and test sets (AUC of 0.87 and 0.57, respectively). The combination of ANOVA and NNET also showed a considerable generalization gap between training and test set, both with and without the inclusion of clinical parameters: the average training AUC was 0.79, while test AUC was 0.61. These results were in line with the radiomic case, where NNET was the model presenting lower stability and generalization.

The selector-classifier pipeline with the highest test performance was the one composed by the CLUSTER reducer and the XGB classifier, without including clinical data: in this case, the final train AUC was 0.70 ± 0.02, while test AUC was 0.63 ± 0.03.

#### Radiomic and deep features

**Figure 6:**
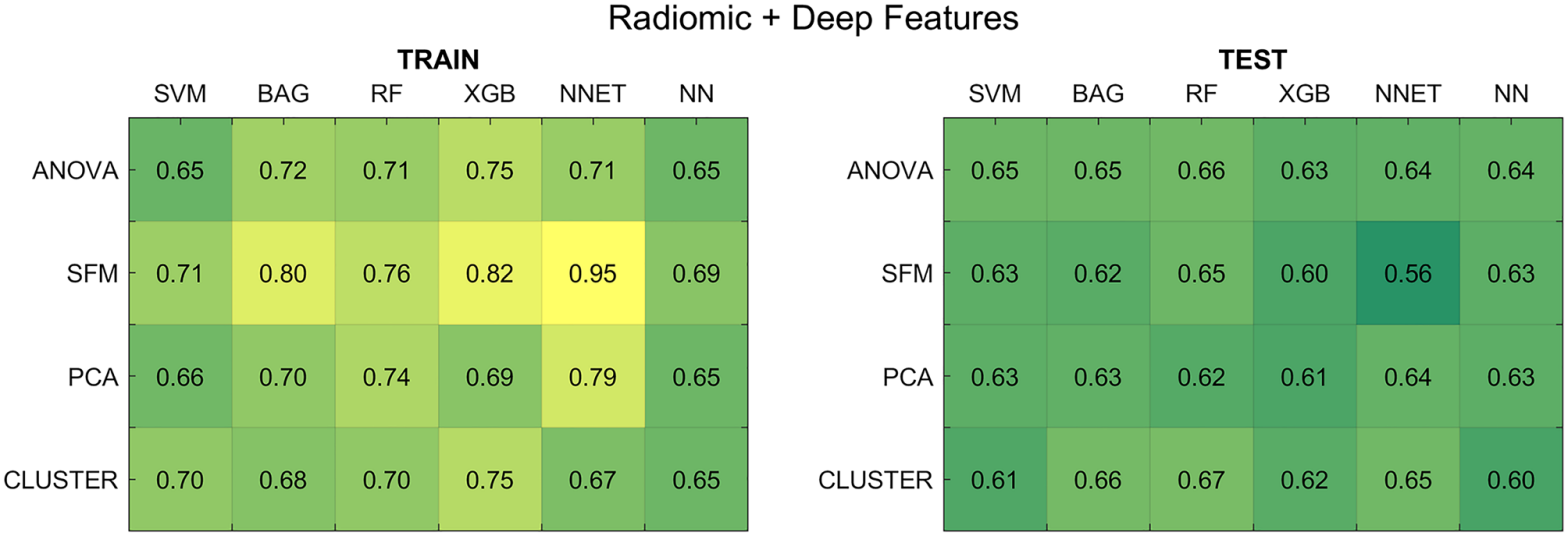
Results for the radiomic and deep feature-based models without including clinical data. (Left panel) Average AUCs on the five training splits. (Right panel) Average AUCs on the test splits. *SFM *SelectFromModel, *PCA* principal component analysis, *CLUSTER* feature agglomeration through clustering, *SVM* support vector machines, *BAG* bagging, *RF* random forest, *XGB* extreme gradient boosting, *NNET* neural network, *NN* k-nearest neighbours.

Figure 6 reports the average AUCs across the five shuffle splits, considering the optimal hyper-parameters for each pipeline, for the case in which clinical data were not considered in the feature set (results including clinical data are reported in Supplementary Fig. S6).

When combining radiomic and deep features, the SFM feature selector showed the highest divergence between training and test set, especially when coupled with NNET (AUC of 0.95 and 0.56, respectively). These results were in line with radiomic and deep cases, where NNET also showed a poor generalization ability.

As for the case of models trained with radiomic and deep features alone, also in this case the inclusion of clinical information did not determine significant improvements in model performance. Globally, results were in line with the radiomic case: the best selector-classifier pipeline, which was the one composed by the CLUSTER reducer and the RF classifier, achieved a final train AUC of 0.70 ± 0.01 and a test AUC of 0.67 ± 0.02.

### Results for CNN-based approach

The 2D-CNN model trained on original slices considering the best hyper-parameters presented good generalization on the test set, achieving a training and test AUC of 0.63. The same model with the inclusion of clinical data, i.e., 2D CNN Cl, showed the highest test performance at the expense of a greater gap between training and test set: AUC equal to 0.69 ± 0.01 and 0.64 ± 0.04, respectively.

The lowest performance was that of the 2.5D-CNN. For this model, the training AUC was equal to 0.66 and the test AUC was 0.61. When including clinical data, the same model achieved training and test AUC of 0.64 and 0.61, respectively. Although this model was trained with higher regularization parameters (i.e., L1, L2 weight decays and dropout) with respect to 2D-CNN to deal with a higher probability of overfitting, it could not handle the increase in architecture complexity and the decrease in the sample size, which resulted in reduced performances both in terms of prediction and generalization capability.

## Discussion

In this work, we implemented and investigated two different approaches for the prediction of the 2-year OS in NSCLC patients from the public LUNG1 dataset, one was feature-based, while the other was CNN-based. For the former approach, at first we built a baseline model considering only clinical data. Secondly, the hand-crafted radiomic features extracted from the three-dimensional GTV ROI and the deep features computed by means of a CAE were fed to 24 different pipelines, obtained as a combination of four feature selection/reduction techniques and six classification methods to predict the 2-year OS. In the latter approach, the classification task was directly accomplished by feeding the images into a CNN and considering several architectures. Each approach was further tested by including clinical parameters either within the feature set or together with the image fed to the CNN, in order to assess whether any of the available clinical information could significantly improve model performance.

For all DL-based methods, we had the limitation of the intrinsic complexity of model training, which required a high computational power and large datasets with respect to radiomics. Therefore, to tackle this limit, we had to restrict the analysis to the implementation of 2D-CAEs and 2D-CNNs, hence reducing the study to a bi-dimensional analysis of the ROI.

The overall analysis has determined some important conclusions. In general, the two approaches showed comparable performance in the prediction of the 2-year OS. Nevertheless, it is worth noticing that the radiomic feature-based approach was able to predict the 2-year OS with a higher test AUC (equal to 0.67 for RF classifier and ANOVA selector) and a lower discrepancy between training and test set performances, compared to DL approaches.

The poor performance of deep feature-based models could be ascribed to the fact that, unlike radiomics, for DL we implemented a bi-dimensional analysis of the lesion, which has determined a loss of spatial information. Indeed, the deep feature-based approach showed the greatest gap between training and test set performance, thereby warning of possible overfitting caused by a suboptimal selection/reduction of the initial deep feature set.

Models trained with merged radiomic and deep features showed better performance compared to deep features alone, but similar to radiomic features. However, compared to the latter, there was a slight improvement for some pipelines (e.g., CLUSTER and RF) and a general performance decrease for others (e.g., SFM and SVM). These results suggest that radiomic features drove model training, hence carrying the most relevant predictive information for the 2-year OS with respect to deep features.

As for clinical data performance, models trained with clinical features alone showed test AUC values fairly close to chance. Instead, when clinical data were coupled with radiomic and/or deep features, the performance of the models remained almost unchanged. This finding seems to suggest that the considered clinical data—available with the LUNG1 dataset—do not significantly contribute to the 2-year OS prediction in NSCLC.

However, this does not exclude that different uses of patients’ clinical information (e.g., to perform other predictive objectives, to stratify patients into NSCLC subtypes and TNM stages^16^) may yield promising predictive results. In future investigations it would also be interesting to test whether other clinical features or prognostic biomarkers (e.g., gene features^27^) may improve survival prediction in NSCLC.

CNN-based classification was not able to exceed a maximum test AUC of 0.64. The reason for this limitation could be due to the high regularization that was mandatory to reduce the risk of overfitting. However, our results are in line with previous findings.

In conclusion, the wide range of methods and approaches investigated in this work produced further evidence that the image-based prediction of the 2-year OS on the LUNG1 dataset is a challenging task, with limited margins of improvements. Nevertheless, the LUNG1 dataset continues to play a pivotal role: possibly, better models could be achieved when focusing on a different outcome, e.g., stage^21^, histochemical type^14^. Moreover, more accurate models for 2-year OS prediction could be built on other datasets, and could still rely on LUNG1 for their external validation^21,26^. This is the advantage of publicly sharing large datasets with the research community^10^.

In future works, it will be interesting to investigate the use of DL approaches for synthetic data augmentation, to apply different DL learning techniques (e.g., contrastive learning), to perform a whole-lung analysis, hence including information from the tissue surrounding the GTV^28^, or to employ three-dimensional DL models, in order to assess whether these methods could achieve better performances, thereby confirming the use of these techniques as a promising non-invasive adjunctive for tissue characterization and prediction of cancer diagnosis and prognosis.

### Supplementary Information

Supplementary Information.

## Data Availability

The dataset analyzed during the current study is available in the TCIA repository^11^ (https://wiki.cancerimagingarchive.net/display/Public/NSCLC-Radiomics).
