# Author Correction: Radiomics and deep learning methods for the prediction of 2-year overall survival in LUNG1 dataset

**DOI:** 10.1038/s41598-023-44197-1

**Published:** 2023-10-16

**Authors:** Anna Braghetto, Francesca Marturano, Marta Paiusco, Marco Baiesi, Andrea Bettinelli

**Affiliations:** 1 Physics and Astronomy Department “Galileo Galilei”, University of Padova, Via Marzolo 8, 35131 Padua, Italy; 2 INFN, Sezione di Padova, Via Marzolo 8, 35131 Padua, Italy; 3 Medical Physics Department, Veneto Institute of Oncology-IOV IRCCS, Padua, Italy; 4 Department of Information Engineering, University of Padova, Padua, Italy

Correction to: *Scientific Reports* 10.1038/s41598-022-18085-z, published online 19 August 2022

The Funding section in the original version of this Article was omitted. The Funding section now reads:

“This work received ‘Ricerca Corrente 2023’ funding from the Italian Ministry of Health to cover publication costs.”

The original Article has been corrected.

