# Supplementary material for: Radiomics and deep learning methods for the prediction of 2-year overall survival in LUNG1 dataset
Source: Sci Rep. 2022 Aug 19;12:14132. doi: 10.1038/s41598-022-18085-z (PMC9391464; doi:10.1038/s41598-022-18085-z)
Supplement: Supplementary file 1 — Supplementary Information. [file 41598_2022_18085_MOESM1_ESM.docx]

**Radiomics and deep learning methods for the prediction of 2-year overall survival in LUNG1 dataset**

**Anna Braghetto**^1,2,†,*^**, Francesca Marturano**^3,†^**, Marta Paiusco**^3^**, Marco Baiesi**^1,2^ **and Andrea Bettinelli**^3,4^

^1^ Physics and Astronomy Department “Galileo Galilei”, University of Padova, via Marzolo 8, 35131, Padova, Italy

^2^ INFN, Sezione di Padova, via Marzolo 8, 35131, Padova, Italy

^3^ Medical Physics Department, Veneto Institute of Oncology - IOV IRCCS, Padova, Italy

^4^ Department of Information Engineering, University of Padova, Padova, Italy

^*^anna.braghetto@phd.unipd.it

^†^these authors contributed equally to this work

**1. RADIOMIC FEATURES EXTRACTION THROUGH PYRADIOMICS SOFTWARE**

**1.1 FEATURE FAMILIES**

| **CLASS** | **NUMBER OF FEATURES** |
| --- | --- |
| Shape | 14 (not considered: compactness 1-2) |
| First order | 18 (not considered: standard deviation) |
| GLCM | 23 (not considered: sum average) |
| GLRLM | 16 |
| GLSZM | 16 |
| GLDM | 14 |
| NGTDM | 5 |

**Table S1.** The 106 Radiomic features that were extracted from the three-dimensional GTV ROIs. (GLCM = gray level co-occurrence matrix; GLRLM = gray level run length matrix; GLSZM = gray level size zone matrix; GLDM = gray level dependence matrix; NGTDM = neighbouring gray-tone difference matrix)

**1.2 FILTER PARAMETERS AND SETTINGS**

| **FILTER** | **NUMBER OF IMAGES** | **SETTINGS** |
| --- | --- | --- |
| Original | 1 | Bin Width = 25 |
| Wavelet: LLL, LLH, LHL, LHH, HHH, HHL, HLH, HLL | 8 | Bin Count = 32 |
| Square root | 1 | Bin Count = 32 |
| Gradient magnitude | 1 | Bin Count = 32 |
| Laplacian of a Gaussian: sigma = 1.0 | 1 | Bin Count = 32 |

**Table S2.** Type of images from which radiomic features are extracted. (L = low pass filter; H = high pass filter; bin Width = width of the bins for fixed-bin size intensity discretization method; bin Count = number of bins for fixed-bin number intensity discretization method)

All the other parameters are set to default ones.

**2. CLASSIFICATION PIPELINES FOR RADIOMIC AND DEEP FEATURE APPROACHES**

**2.1 FEATURE SELECTION/REDUCTION METHODS**

*ANOVA* is a statistical test of variance that studies the difference between group means. In this context, it analyses the data by comparing both intra- and inter-group variability[^1^](https://www.zotero.org/google-docs/?8H1bRL). The selection is performed by ranking the F-values and by choosing the first K features, where K is a user-defined parameter

*SelectFromModel (SFM)* feature selection method studies feature importance by training a random forest model on the initial set of features. A random forest is a supervised learning classifier that is fed with all the features in order to predict the outcome (e.g., the 2-year overall survival). After the training phase, feature selection is performed by ranking feature importance and by choosing the first K features.

*Principal component analysis (PCA)* is a statistical analysis method that extracts the K components that explain most of the variance in the data. The computation of the principal components is carried out by singular value decomposition of the original matrix of features. The K largest singular values are used to map the original D−dimensional data into K-dimensional data (with K <= D), hence reducing the number of features[^2^](https://www.zotero.org/google-docs/?FolGV6).

The *clustering method (CLUSTER)* is an unsupervised learning technique that reduces data dimension by hierarchically merging similar features into a cluster. The process is reiterated until the desired number of clusters is achieved[^3^](https://www.zotero.org/google-docs/?Z0Dtqv).

| **SELECTOR/REDUCER** | **K** |
| --- | --- |
| ANOVA | [5, 10, 20, 40] |
| SFM | [5, 10, 20, 40] |
| PCA | [5, 10, 20, 40] |
| CLUSTER | [5, 10, 20, 40] |

**Table S3.** Method for feature selection/reduction and the corresponding hyperparameters.

**2.2 CLASSIFIERS**

*Support vector machines* (SVM)[^4,5^](https://www.zotero.org/google-docs/?zCcAiK) are supervised learning models that are used both for classification and regression tasks. Classification is achieved by mapping the input data into high-dimensional spaces, through the definition of a hyperplane that divides the data into classes by imposing maximum distance among them.

*Bagging* (BAG)[^6^](https://www.zotero.org/google-docs/?GliqOB) is an ensemble learning algorithm that performs supervised classification and regression tasks. The input dataset is divided into subsets that are independently used as training sets. Then the outputs of each subset are aggregated for the final prediction. When the dataset is small, the alternative is using the bootstrapping method that resamples the original dataset with replacement. For regression problems, subset predictions are combined by averaging, while for classification problems by voting.

*Random forest* (RF)[^6,7^](https://www.zotero.org/google-docs/?bcAQPd) is an ensemble learning algorithm for supervised classification and regression tasks. In the training phase, the method builds several decision trees that perform the prediction task independently based on random subsets of input features. The final output is the weighted predictions of all trees (for regression) or the class predicted by most trees (for classification).

*Extreme gradient boosting* (XGB)[^6^](https://www.zotero.org/google-docs/?UUjACD) is a supervised learning model that combines gradient descent and boosting algorithms to build ensembles of decision trees. In gradient boosting, each decision tree is added subsequently to the ensemble in order to move the cost function in the opposite direction of its gradient. In extreme gradient boosting, it is also added a regularisation term that allows to faster minimize the loss and to build a stronger classier.

*Neural networks* (NNET)[^6,8^](https://www.zotero.org/google-docs/?51lzTx) are supervised learning models used both for regression and classification tasks. They emulate the brain structure and are made up of connected units, called neurons. In this context, a neural network has several layers of neurons that are connected through links with a specific weight learned in the training phase. A generic layer of neurons takes in input a vector of features which is weighted and fed to a nonlinear activation function that returns a scalar outcome.

*K−nearest neighbors* (NN) is a supervised learning method used to classify data based on their pattern. For each test sample, the task is achieved by considering the K training samples closest to it, while the output depends on whether the algorithm is used for regression (output is a property value) or classification (output is the class given by neighbors vote) problems[^9^](https://www.zotero.org/google-docs/?pMjCCI).

| **CLASSIFIER** | **HYPERPARAMETER** | **VALUES** |
| --- | --- | --- |
| SVM | C  Kernel | 10^-2^, 10^-1^, 10^1^, 10^2^  Linear, Polynomial, Radial Basis Function,  Sigmoid |
| BAG | Number of trees  Maximum depth  Maximum samples | 50, 100  1, 2, 3  0.1, 0.5, 1.0 |
| RF | Number of trees  Minimum samples split  Maximum depth | 50, 100  0.1, 0.5  1, 2, 3 |
| XGB | Number of trees  Minimum child weight  Maximum depth  Learning rate | 50, 100  10, 15  1, 2, 3  0.01, 0.005 |
| NNET | Architecture  Regularisation L2 | (8), (16), (8,8), (16,16)  10^0^, 10^-1^ |
| NN | Neighbours  Weight function | 50, 100, 200  Uniform |

**Table S4.** Classifiers and the corresponding hyperparameters. (SVM = Support Vector Machines; BAG = Bagging; RF = Random Forest; XGB = Extreme Gradient Boosting; NNET = Neural Network; NN = k-Nearest Neighbours).

**3. DEEP LEARNING MODELS**

**3.1 CONVOLUTIONAL AUTOENCODER (CAE)**


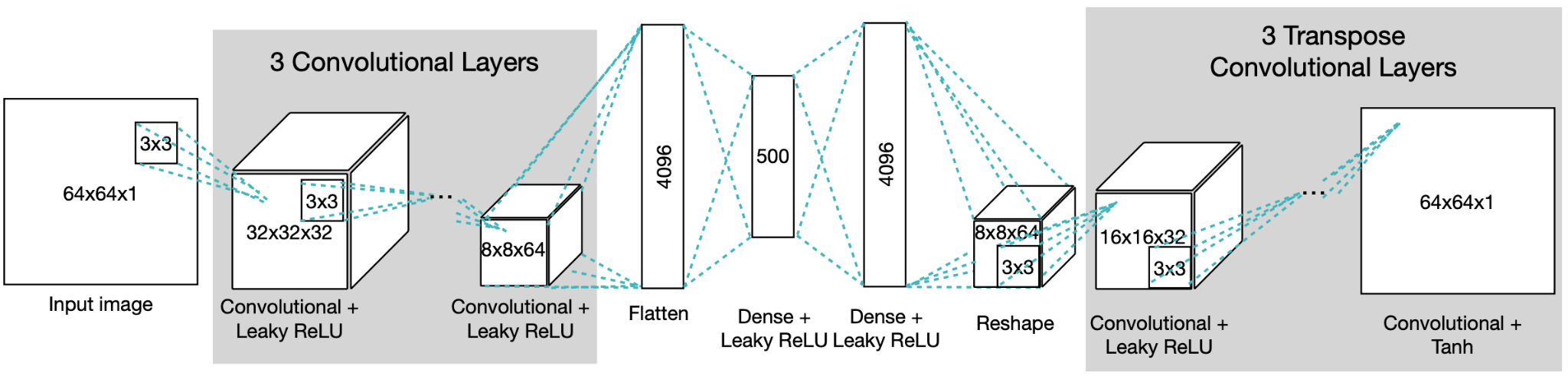


**Figure S1.** Structure of a Convolutional Autoencoder (CAE). The input image is fed to the encoder that compresses the image into a set of 500 latent variables which synthesise the whole information in the image. The variables are then fed to the decoder that decompresses them into the output image. During the training phase, the whole structure of the CAE is optimised to obtain an output image as similar as possible to the input one.

| **HYPERPARAMETER** | **VALUE** |
| --- | --- |
| Activation function after convolutional and dense layers | Leaky ReLU |
| Activation function output | Tanh |
| Weight initialization | Glorot Uniform |
| Optimizer | Adam |
| Loss | Mean Squared Error |

**Table S5.** CAE hyperparameters fixed in the grid search.

| **HYPERPARAMETER** | **VALUES** |
| --- | --- |
| Learning rate | 10^-5^, 10^-4^ |
| Weight decay L2 | 10^-6^, 10^-5^ |

**Table S6.** CAE hyperparameters tuned in the grid search.

**3.2 CONVOLUTIONAL NEURAL NETWORK (CNN)**

**
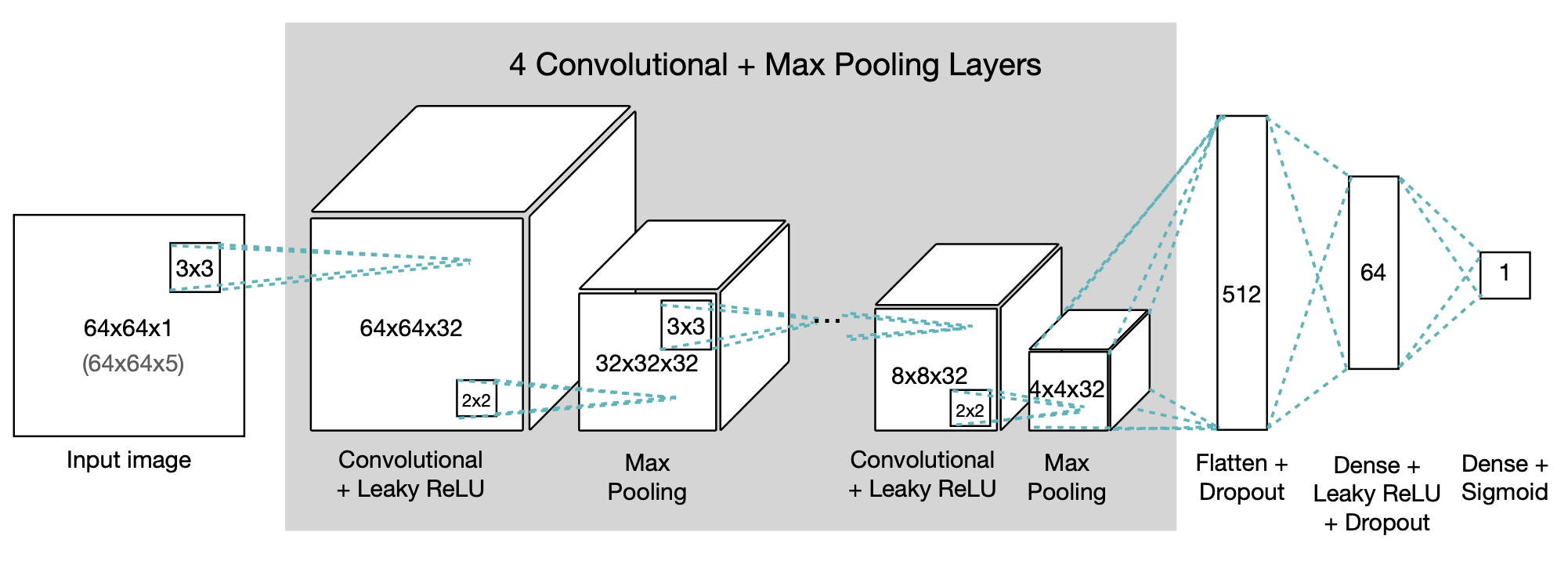
**

**Figure S2.** Structure of a 2D (or 2.5D, in grey) convolutional neural network (CNNs) applied to the bi-dimensional image slices. The input slice (for 2D) or image (for 2.5D) is fed to the convolutional layer that computes a set of feature maps that are then passed to the pooling layer to reduce their dimension. The process is repeated 4 times. Then, the resulting sample is flattened into a vector and fed to a feed-forward layer for the final prediction of the outcome. During the training procedure, the whole structure is optimized to predict the outcome.

| **HYPERPARAMETER** | **VALUE** |
| --- | --- |
| Activation function after convolutional and dense layer | Leaky ReLU |
| Activation function output | Sigmoid |
| Weight initialization | Glorot Normal |
| Learning rate | 10^-5^ |
| Optimizer | Adam |
| Loss | Binary cross entropy |

**Table S7.** 2D-CNN and 2.5D-CNN hyperparameters fixed in the grid search.

| **HYPERPARAMETER** | **VALUES** |
| --- | --- |
| Weight decay L1 | 5x10^-4^, 10^-3^ |
| Weight decay L2 | 5x10^-4^, 10^-3^ |
| Dropout rate | 0.25, 0.50 |

**Table S8.** 2D-CNN hyperparameters tuned in the grid search.

| **HYPERPARAMETER** | **VALUES** |
| --- | --- |
| Weight decay L1 | 10^-3^, 2x10^-3^ |
| Weight decay L2 | 10^-3^, 2x10^-3^ |
| Dropout rate | 0.40, 0.60 |

**Table S9.** 2.5D-CNN hyperparameters tuned in the grid search.

To include clinical data in the model, a vector containing the individual clinical parameters was appended to the flatten output of the last convolutional and pooling layer. The whole feature vector is then fed to the feed-forward layer for the final prediction.


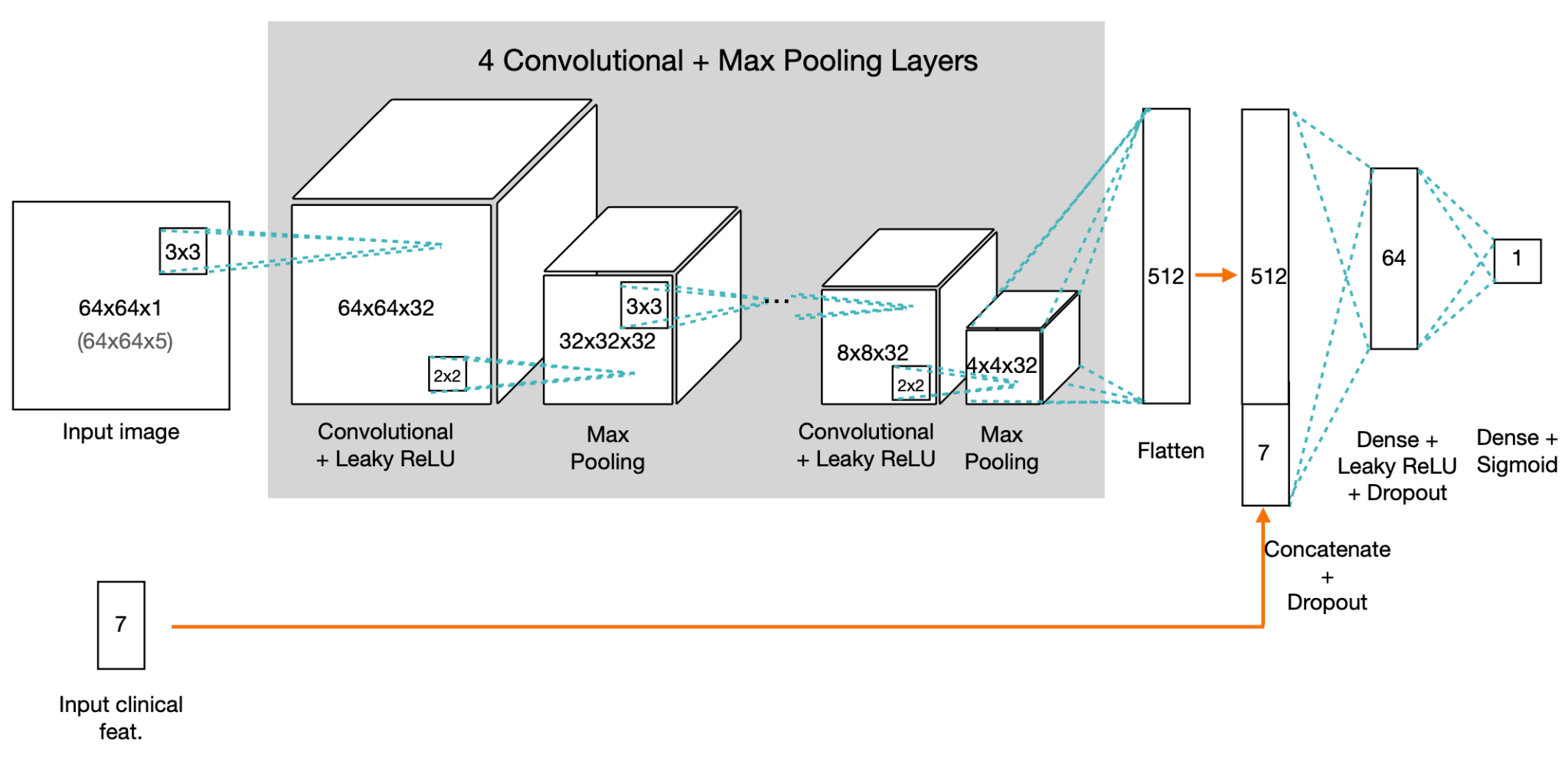


**Figure S3.** Structure of a 2D (or 2.5D, in grey) convolutional neural network (CNNs) applied to the bi-dimensional image slices including clinical data.

**4. CLASSIFICATION RESULTS INCLUDING CLINICAL DATA**

**
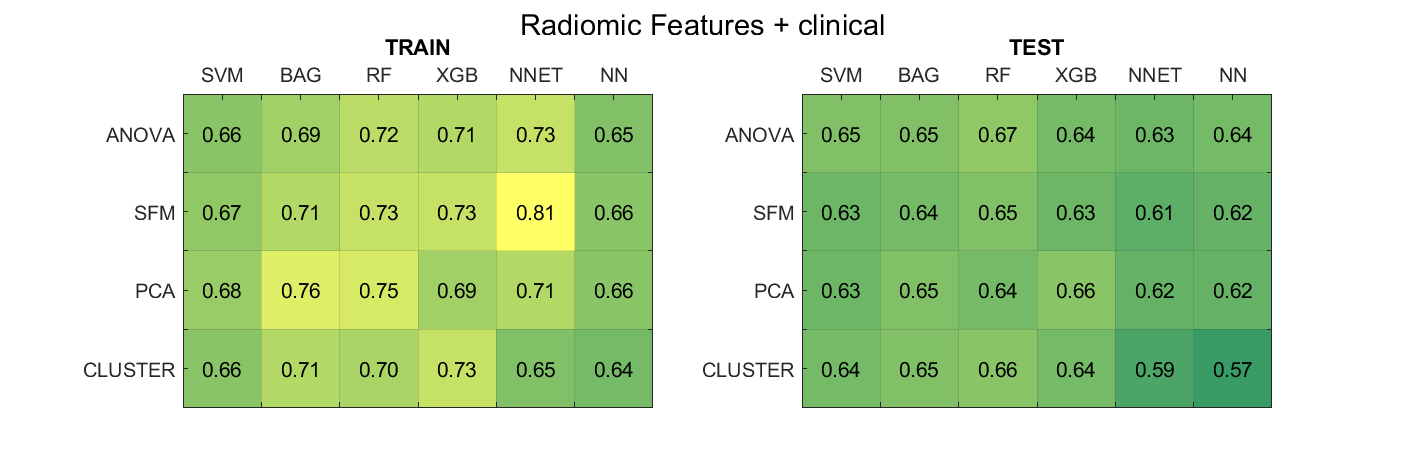
**

**Figure S4.** Results for the radiomic feature-based models with the inclusion of clinical data within the feature set. (Left panel) Average AUCs on the five training splits. (Right panel) Average AUCs on the test splits. (SFM = SelectFromModel; PCA = Principal Component Analysis; Cluster = feature agglomeration through clustering; SVM = Support Vector Machines; BAG = Bagging; RF = Random Forest; XGB = Extreme Gradient Boosting; NNET = Neural Network; NN = k-Nearest Neighbours).

**
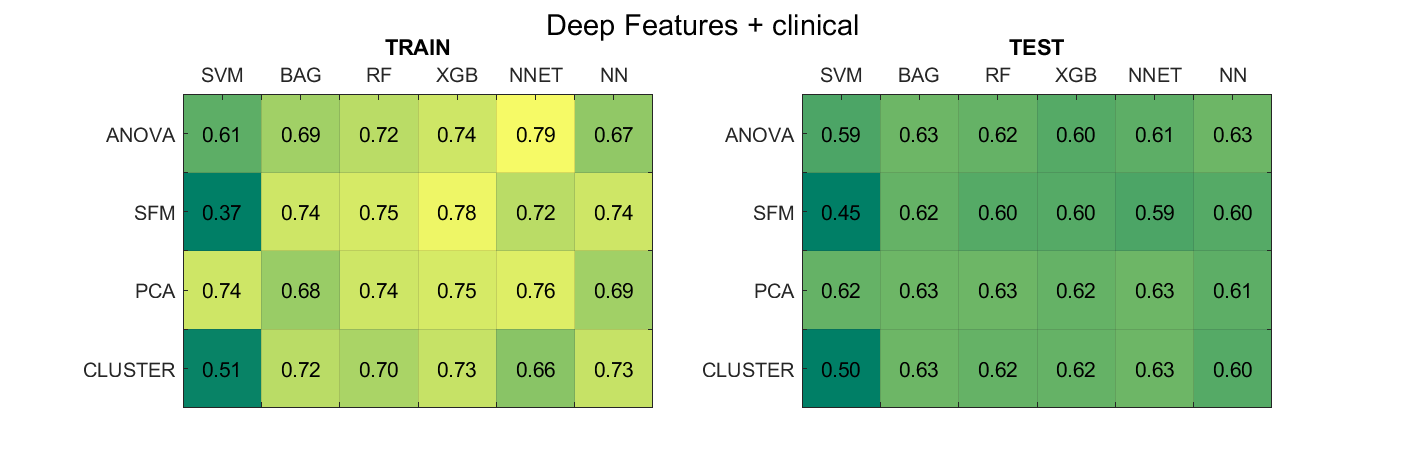
**

**Figure S5.** Results for the deep feature-based models with the inclusion of clinical data within the feature set. (Left panel) Average AUCs on the five training splits. (Right panel) Average AUCs on the test splits. (SFM = SelectFromModel; PCA = Principal Component Analysis; Cluster = feature agglomeration through clustering; SVM = Support Vector Machines; BAG = Bagging; RF = Random Forest; XGB = Extreme Gradient Boosting; NNET = Neural Network; NN = k-Nearest Neighbours).

**
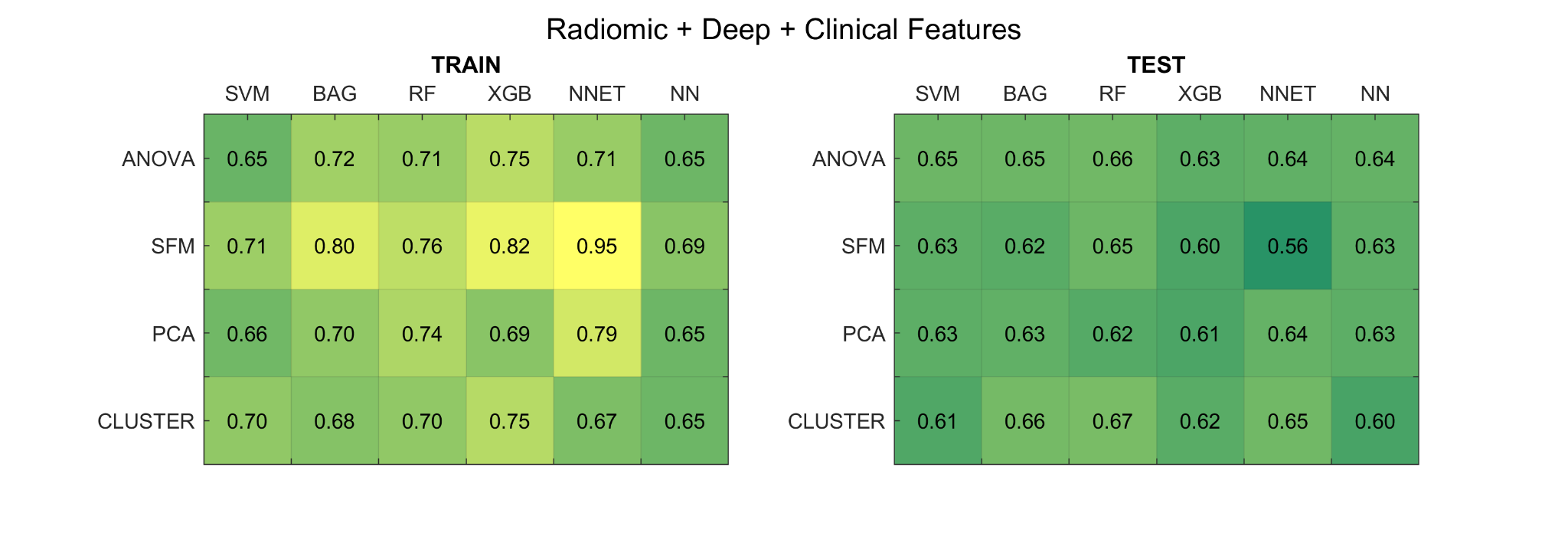
**

**Figure S6.** Results for the radiomic and deep feature-based models with the inclusion of clinical data within the feature set. (Left panel) Average AUCs on the five training splits. (Right panel) Average AUCs on the test splits. (SFM = SelectFromModel; PCA = Principal Component Analysis; Cluster = feature agglomeration through clustering; SVM = Support Vector Machines; BAG = Bagging; RF = Random Forest; XGB = Extreme Gradient Boosting; NNET = Neural Network; NN = k-Nearest Neighbours).

**REFERENCES**

[1. Sawyer, S. F. Analysis of Variance: The Fundamental Concepts. (2009) doi:10.1179/jmt.2009.17.2.27E.](https://www.zotero.org/google-docs/?LqK0I1)

[2. Principal component analysis - Abdi - 2010 - WIREs Computational Statistics - Wiley Online Library. https://wires.onlinelibrary.wiley.com/doi/full/10.1002/wics.101.](https://www.zotero.org/google-docs/?LqK0I1)

[3. Omran, M. G. H., Engelbrecht, A. P. & Salman, A. An overview of clustering methods. *Intell. Data Anal.* **11**, 583–605 (2007).](https://www.zotero.org/google-docs/?LqK0I1)

[4. Hearst, M. A., Dumais, S. T., Osuna, E., Platt, J. & Scholkopf, B. Support vector machines. *IEEE Intell. Syst. Their Appl.* **13**, 18–28 (1998).](https://www.zotero.org/google-docs/?LqK0I1)

[5. Cortes, C. & Vapnik, V. Support-vector networks. *Mach. Learn.* **20**, 273–297 (1995).](https://www.zotero.org/google-docs/?LqK0I1)

[6. Mehta, P. *et al.* A high-bias, low-variance introduction to Machine Learning for physicists. *Phys. Rep.* **810**, 1–124 (2019).](https://www.zotero.org/google-docs/?LqK0I1)

[7. Ali, J., Khan, R., Ahmad, N. & Maqsood, I. Random Forests and Decision Trees.](https://www.zotero.org/google-docs/?LqK0I1)

[8. Rumelhart, D. E., Widrow, B. & Lehr, M. A. The basic ideas in neural networks. *Commun. ACM* **37**, 87–93 (1994).](https://www.zotero.org/google-docs/?LqK0I1)

[9. Cunningham, P. & Delany, S. J. k-Nearest Neighbour Classifiers. (2007).](https://www.zotero.org/google-docs/?LqK0I1)
